# Investigating the hypothermic effects of fluoroquinolone antimicrobials on non-bacterial fever model mice

**DOI:** 10.1186/s40780-024-00392-4

**Published:** 2024-11-04

**Authors:** Ryohei Hara, Kazuaki Taguchi, Hiromi Ogino, Yuko Okamoto, Yuki Enoki, Junko Kizu, Seiji Hori, Kazuaki Matsumoto

**Affiliations:** 1 Division of Pharmacodynamics, Faculty of Pharmacy, Keio University, 1-5-30 Shibakoen, Minato-Ku, Tokyo, 105-8512 Japan; 2 Division of Practical Pharmacy, Faculty of Pharmacy, Keio University, Tokyo, Japan; 3 Department of Infectious Diseases and Infection Control, Jikei University School of Medicine, Tokyo, Japan

**Keywords:** Fluoroquinolone, Hypothermia, Fever, Mice, Glucocorticoid

## Abstract

**Background:**

Fluoroquinolone (FQ) antimicrobials have antipyretic effects during the treatment of bacterial infections; however, it is not clear whether these are due to their antimicrobial activities or their hypothermic effects. In this study, we investigated the hypothermic effects of FQ antimicrobials (ciprofloxacin [CPFX], gatifloxacin [GFLX], and levofloxacin [LVFX]) on fever by evaluating rectal body temperature changes in a mouse model of non-bacterial fever.

**Methods:**

CPFX, GFLX, and LVFX were administered intraperitoneally to non-bacterial fever model mice induced by yeast. Rectal body temperature was measured up to 180 min after administration.

**Results:**

A decrease in rectal body temperature of up to 1.2 °C for CPFX, 3.4 °C for GFLX, and 1.0 °C for LVFX was observed. The decrease in temperature was induced by an increase in the plasma concentration of FQ antimicrobials, suggesting that they are responsible for the temperature reduction. Focusing on glucocorticoids, one thermoregulation mechanism, we investigated the substances responsible for the reduction in rectal body temperature induced by FQ antimicrobials. Aminoglutethimide (an inhibitor of glucocorticoid production) were premedicated, followed by intraperitoneal administration of GFLX in the yeast-induced fever mouse model, resulting in attenuated GFLX-induced hypothermic effects.

**Conclusions:**

These results suggest that certain antipyretic effects of CPFX, GFPX, and LVFX during fever may contribute to their hypothermic effects; certain mechanisms are glucocorticoid-mediated.

## Background

Fluoroquinolone (FQ) antimicrobials have a broad antibacterial spectrum and strong antibacterial activity; currently, 15 types are used in Japan and more than 40 are used worldwide to treat various bacterial infections, including urinary tract infections and pneumonia. In addition to their antibacterial activities, FQ antimicrobials have been reported to possess various bioactivities, including immunomodulatory and anti-inflammatory effects [1–3], and are expected to have additional impacts against various infections. Takasuna et al. reported that levofloxacin (LVFX) administration to healthy rabbits decreased body temperature [4]. Miyazaki et al. reported that gatifloxacin (GFLX), ciprofloxacin (CPFX), and LVFX induced similar decreases in body temperature in healthy mice [5]. These reports suggest that the antimicrobial activities of FQ antimicrobials as well as their hypothermic effects may contribute to fever reduction during bacterial infections; however, the contribution of these effects is not clear. In studies using bacterially induced animal fever models, it is difficult to directly evaluate the hypothermic effects of FQ antimicrobials because antipyretic effects occur as a result of their antimicrobial effects. To exclude the antipyretic effects of FQ antimicrobials, we investigated the effects of FQ antimicrobials (CPFX, GFLX, and LVFX) on body temperature during fever in non-bacterial fever model mice induced with yeast, which is commonly used to evaluate antipyretic effects of substances [6–8]. We investigated the mechanism of their hypothermic effects, focusing on glucocorticoids, which are associated with decreased body temperature along with other drugs (ipsapirone) [9].

## Methods

### Animals

Male mice (ICR strain, 5 weeks old, Sankyo Lab Service Co., Japan) were maintained until 6 weeks of age in a specific pathogen-free room with free access to water and food until the experiment was conducted.

### Measurement of mouse rectal body temperature

After the rectal body temperature was confirmed to be at 37 °C or higher, 20% dried yeast saline suspension (Tanabe Mitsubishi Pharma Corporation, Tokyo, Japan) at 50 mL/kg was administered subcutaneously in the dorsal neck. Rectal body temperature was measured again 18 h after administration of the dried yeast solution, and mice with a fever of 0.8 °C or higher compared to their temperature before dried yeast solution administration were considered to be non-bacterial fever model mice. CPFX (LKT Laboratory, St. Paul, MN, USA), GFLX (LKT Laboratory), and LVFX (Tokyo Kasei Kogyo Co., Ltd., Tokyo, Japan) were intraperitoneally administered to yeast-induced fever model mice. The dose of CPFX and GFLX was adjusted to 100, 50, and 25 mg/kg (5 mL/kg) in saline solution. The dose of LVFX was adjusted to 100 and 50 mg/kg (5 mL/kg) in saline solution. The rectal body temperature was measured using a modified version of the method described by Ono et al. [10]. Briefly, each mouse was placed loosely in a small cloth bag, and a thermometer (MT-132, Mother Tool, Ueda, Japan) was inserted 25 mm into the rectum to measure body temperature every 6 min for 180 min after the administration of each FQ antimicrobial. Mice in the control group were intraperitoneally administered saline, and rectal body temperature was measured in the same manner. To avoid the anesthetic influence on body temperature, these manipulations were performed under unanesthetized conditions.

### Evaluation of plasma concentrations of FQ antimicrobials in yeast-induced fever model mice

CPFX (100 mg/kg, 5 mL/kg), GFLX (100 mg/kg, 5 mL/kg), and LVFX (100 mg/kg, 5 mL/kg) were administered intraperitoneally to yeast-induced fever model mice. At 10, 20, 30, 45, 60, 90, 120, and 180 min after administration, mice were euthanized, and blood samples were collected. Blood samples were immediately centrifuged (4 °C, 3000 rpm, 10 min), and the resulting plasma FQ antimicrobial concentration was determined using high-performance liquid chromatography. The equipment and measurement conditions used were as follows: Pump, L-7100 (Hitachi, Ltd.); column oven unit, L-7300 (manufactured by Hitachi, Ltd.); sample injector, MICROLITER #705 (Kyowa Seimitsu Co., Ltd.); autosampler, L-2200 (Hitachi, Ltd.); pre-column, TSKguardgel ODS-80TM (7 mm, Tosoh Corp.); column, TSK-GEL ODS-80TM (250 mm × 4.6 mm i.d., 7 mm, Tosoh); fluorescence detector, L-7485 (Hitachi, Ltd.); column temperature, 23 °C; flow rate, 1 mL/min; mobile phase A, 10 mM sodium dodecyl sulfate (Nacalai Tesque Co., Kyoto, Japan) + 10 mM tetrabutylammonium acetate (Sigma Chemical Co., St. Louis, MO, USA) + 25 mM citric acid (Nacalai Tesque Co., Kyoto, Japan); mobile phase B, acetonitrile (Nacalai Tesque Co., Kyoto, Japan) (A:B = 57:43, v/v); wavelength (excitation/fluorescence), 280 nm/450 nm.

### Rectal body temperature in yeast-induced fever model mice during preadministration of aminoglutethimide (AMG)

AMG (Sigma Chemical Co., St. Louis, MO, USA, 25 mg/kg, 5 mL/kg) solution was administered intraperitoneally to yeast-induced fever model mice. Sixty minutes later, GFLX (100 mg/kg, 5 mL/kg) was administered intraperitoneally, and the rectal body temperature was measured every 6 min until 180 min after GFLX administration.

### Statistical analysis

All data are expressed as mean ± S.E. Dunnett's test was employed to analyse the significance of changes in rectal body temperature over time after intraperitoneal administration of FQ antimicrobials. Regarding the significance analysis, one-way ANOVA with the Bonferroni test was used to examine changes in rectal body temperature following glucocorticoid production inhibitor. A two-tailed difference was considered significant (P < 0.05). SPSS version 29.0 for Windows (IBM Corp., Armonk, NY, USA) was used for statistical analyses.

## Results

### Rectal body temperature changes of FQ antimicrobials in yeast-induced fever model mice

**Fig. 1:**
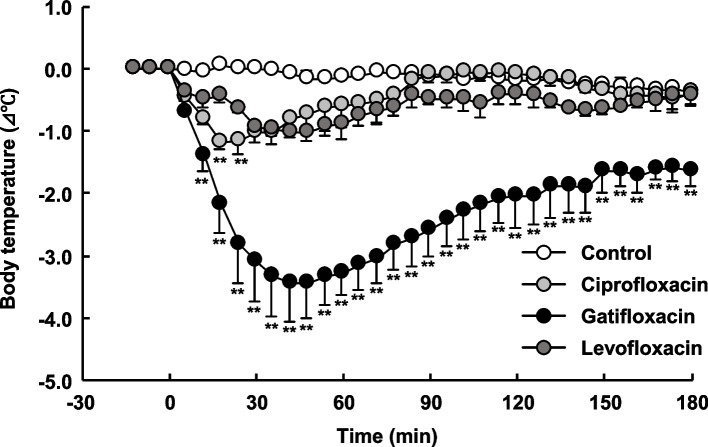
Rectal body temperature changes over time following intraperitoneal administration of ciprofloxacin, gatifloxacin, and levofloxacin at 100 mg/kg in yeast-induced fever model mice. Each point represents the mean ± S.E. of the change in rectal body temperature from baseline in each mouse (*n* = 3). Significant differences were determined using Dunnett's multiple comparison test. **p < 0.01 vs. 0 min of each fluoroquinolone antimicrobial

Rectal body temperature changes in yeast-induced fever model mice when each FQ antimicrobial was administered at 100 mg/kg are shown in Fig. 1. A decrease in rectal body temperature was observed 10 min after administration of all FQ antimicrobials, with a maximum decrease of 1.2 °C for CPFX, 3.4 °C for GFLX, and 1.0 °C for LVFX. The decrease in the rectal body temperature with CPFX and LVFX was transient and returned to a value similar to that of the control group 180 min after administration; in contrast, GFLX administration resulted in a decrease in rectal temperature of 1.6 °C that persisted even after 180 min of administration. No significant decrease in rectal body temperature was observed at doses of 50 mg/kg or less for CPFX and LVFX, or at 25 mg/kg or less for GFLX (Table 1).

**Table 1:** Rectal body temperature changes over time following intraperitoneal administration of ciprofloxacin, gatifloxacin, and levofloxacin at dose-dependent manner in a mouse model of non-bacterially induced fever

	Dose (mg/kg)	Time after fluoroquinolone injection (min)					
		12	18	30	60	120	180
Ciprofloxacin	100	-0.8	-1.2	-1.0	-0.6	-0.1	-0.4
		± 0.1^*^	± 0.1^**^	± 0.2^**^	± 0.4	± 0.1	± 0.2
	50	-0.6	-0.4	-0.3	-0.4	-0.4	-0.7
		± 0.2	± 0.2	± 0.2	± 0.2	± 0.1	± 0.1
	25	-0.2	-0.1	0.0	-0.1	-0.2	-0.6
		± 0.2	± 0.2	± 0.1	± 0.1	± 0.2	± 0.3
Gatifolxacin	100	-1.4	-2.2	-3.1	-3.3	-2.0	-1.6
		± 0.2^**^	± 0.5^**^	± 0.7^**^	± 0.3^**^	± 0.5^**^	± 0.3^**^
	50	-0.5	-0.7	-0.8	-0.8	-0.5	-0.7
		± 0.1^*^	± 0.1	± 0.2	± 0.1	± 0.1	± 0.1
	25	-0.1	-0.2	-0.2	-0.2	-0.2	-0.6
		± 0.0	± 0.1	± 0.1	± 0.1	± 0.2	± 0.2
Levofloxacin	100	-0.5	-0.4	-0.9	-0.9	-0.4	-0.4
		± 0.2	± 0.1^*^	± 0.1^**^	± 0.3^*^	± 0.2	± 0.2
	50	-0.1	-0.1	0.0	0.0	-0.3	-0.7
		± 0.1	± 0.1	± 0.2	± 0.2	± 0.1	± 0.1

Each value represents means ± S.E of value (degree) of rectal temperature decreased from initial rectal temperature in each mouse

^*^P < 0.05, ^**^P < 0.01 vs. 0 min

### Plasma concentrations of FQ antimicrobials in yeast-induced fever model mice

**Fig. 2:**
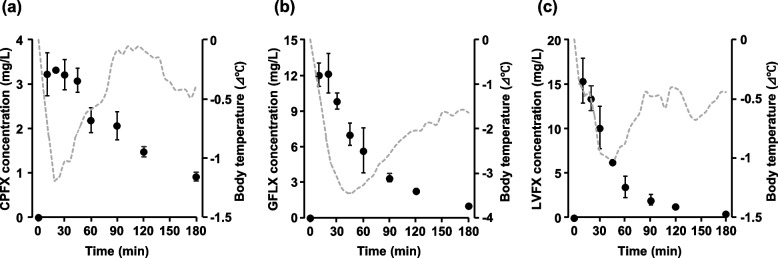
Plasma concentrations of **a** ciprofloxacin (CPFX), **b** gatifloxacin (GFLX), and **c** levofloxacin (LVFX) administered intraperitoneally at 100 mg/kg in yeast-induced fever model mice. Each point represents the mean blood concentration of fluoroquinolone antimicrobials in each mouse ± S.E. (*n* = 3). Dotted line: rectal body temperature changes over time, shown in Fig. 1

The plasma concentrations and corresponding changes in rectal body temperature when 100 mg/kg of each FQ antimicrobial was administered are shown in Fig. 2. Plasma concentrations of each FQ antimicrobial quickly peaked and then gradually declined to less than 1 μg/mL at 180 min. In addition, the rectal body temperatures of mice decreased with increasing plasma concentrations. CPFX and LEVX-treated mice recovered rectal body temperature as their plasma concentrations decreased (Fig. 2a, c), whereas GFLX showed a decrease in rectal body temperature of 1.6 °C even 180 min after administration when its plasma concentration had decreased below 1 μg/mL (Fig. 2b).

### Effect of glucocorticoids on the decrease in rectal body temperature in yeast-induced fever model mice

**Fig. 3:**
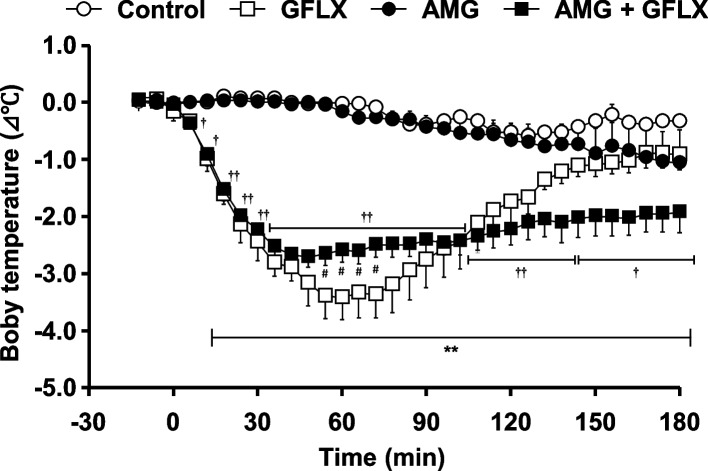
Rectal body temperature changes over time following intraperitoneal administration of gatifloxacin (GFLX) in yeast-induced fever model mice pre-administered aminoglutethimide (AMG). Each point is the mean ± S.E. of the change in rectal body temperature from baseline in each mouse (*n* = 3–5). Statistical differences were assessed by one-way ANOVA followed by the Bonferroni test. **p < 0.01 vs. control. # *p* < 0.05 vs. GFLX. †*p* < 0.05, †† < 0.01 vs. AMG. Changes in rectal body temperature over time for GFLX are shown in Fig. 1

GFLX, which significantly decreased rectal body temperature (Fig. 1), was used to investigate the mechanism underlying the hypothermic effects of FQ antimicrobials during fever. Administration of a glucocorticoid production inhibitor (AMG) to mice did not cause a significant reduction in rectal body temperature (Fig. 3). In contrast, pre-administration of AMG 60 min before GFLX administration suppressed the decrease in rectal temperature induced by GFLX administration.

## Discussion

In this study, the decrease in rectal temperature after administration of CPFX, LVFX, or GFLX was induced by an increase in their plasma concentrations (Fig. 2), suggesting that these FQ antimicrobials decrease rectal temperature by directly or indirectly acting on thermoregulatory mechanisms. Body temperature is regulated both centrally and distally, separated by the blood–brain barrier (BBB) [11]. In the central nervous system, thermoregulation is mediated by gamma-aminobutyric acid (GABA)-ergic EP3-expressing neurons in the preoptic area that send homeostatic GABA-ergic inhibitory signals from the preoptic area to the raphe nucleus [12]. FQ antimicrobials have been shown to inhibit GABA receptors, with CPFX, LVFX, and GFLX having the strongest inhibitory effects [13]. In addition, FQ antimicrobials that act on GABA receptors in the central nervous system must cross the BBB; studies in rats have shown that the brain/blood concentration ratios of CPFX, LVFX, and GFLX 30 to 60 min after administration are approximately 0.1 [14], 0.1 [15], and 0.34–0.58 [16], respectively. Taken together, these reports suggest that CPFX, LVFX, and GFLX may migrate to the brain in small amounts and cause a decrease in rectal body temperature by acting on GABA receptors in the central nervous system. However, since there was no relationship between the degree of temperature reduction of each FQ antimicrobial identified in this study and the brain migration of FQ antimicrobials [14–16] or the strength of GABA receptor inhibitory activity [13], the contribution of rectal body temperature reduction induced by their action on central thermoregulatory mechanisms is likely to be minimal.

Hormone-mediated thermoregulation is one of the distal thermoregulatory mechanisms in humans and animals [17–19]. Glucocorticoids are known to increase blood concentrations and decrease body temperature during fever [20, 21]. In this study, the pre-administration of glucocorticoid production inhibitors reduced the GFLX-induced decrease in rectal body temperature, but the GFLX-induced temperature reduction was not entirely suppressed by the inhibition of glucocorticoid production (Fig. 3). The result suggests that the glucocorticoid-mediated thermoregulatory mechanism partially contributes to the reduction in rectal body temperature induced by FQ antimicrobials during fever. Various factors such as immune cells (macrophages and lymphocytes), PGE2, EP3 receptor-expressing nerves, GABA, and brown adipose tissue are involved in the mechanism of fever in the non-bacterial fever mouse model including yeast-induced fever model [22, 23]. Overall, FQ antimicrobials are responsible for the reduction in rectal body temperature during fever; however, they do so indirectly through a number of variables that regulate thermoregulation, such as glucocorticoids, rather than directly affecting the thermoregulatory systems.

## Conclusions

During fever, CPFX, LVFX, and GFLX decreased rectal body temperature through their inherent hypothermic effects, separate from the antipyretic impact associated with their antibacterial effects. The mechanism of action discovered suggested that several thermoregulatory variables, including glucocorticoids, mediated the effect of FQ antimicrobials on rectal body temperature reduction rather than directly acting on thermoregulatory systems. These findings illustrate one of the diverse bioactivities of FQ antimicrobials.

## Data Availability

All data generated or analysed during this study are included in this published article.

## Abbreviations

FQ: Fluoroquinolone
CPFX: Ciprofloxacin
GFLX: Gatifloxacin
LVFX: Levofloxacin
AMG: Aminoglutethimide
BBB: Brain barrier
GABA: Gamma-aminobutyric acid
